# Results of minimally invasive approach for rheumatic mitral valve disease

**DOI:** 10.1186/1749-8090-10-S1-A240

**Published:** 2015-12-16

**Authors:** Vivek Kanhere, Nikhil Pendse, Vinod Narkhede

**Affiliations:** 1 Department of Cardiothoracic Surgery, Chirayu Medical College & Hospital, Bhopal, Madhya Pradesh, 462030 India; 2 Department of Community Medicine, Chirayu Medical College & Hospital, Bhopal, Madhya Pradesh, 462030 India

## Background/Introduction

The rapid development and refinement of techniques over the past decade have led to the realization that a Minimally invasive (MI) approach enables complex (open) valve surgery to be performed with results equivalent to those of conventional valve surgery done in experienced center. Rheumatic Heart Disease (RHD) continues to be endemic in developing countries like India.

## Aims/Objectives

To compare minimally invasive surgery with standard sternotomy procedure among patients of rheumatic mitral valve disease.

## Method

A retrospective comparative study was carried out in tertiary care hospital of central India. From April 2013 to December 2014, a total of 128 eligible study subjects (45 males and 83 females) [range 7 to 66 years] presented to CVTS department of our hospital were included. Majority of were in NYHA III (52.9%) and NYHA IV (29.7%). All study subjects were considered for minimally invasive (MI) approach, however only 63 study subjects fulfilled the eligibility criteria for the above procedure. Clamp time (CT), Cardiopulmonary bypass (CPB) time, intensive care unit (ICU) stay and hospital stay was observed among study subjects. These results were compared to 63 age and sex matched study subjects from 2012 and also with 65 study subjects who had not undergone minimally invasive surgery from the same period.

## Results

Among MI surgery, operative mortality was 02 (3%), re-exploration were 3 (5%) and 3 (5%) re-admission. 01 re-admission was for delayed tamponade and 02 were for non-compliance with oral anticoagulants. Statistically significant lower CT, CPB time and ICU stay was found in minimally invasive surgery when compared with standard sternotomy approach (p < 0.006).

## Discussion/Conclusion

Minimally invasive approach for rheumatic valve yields similar morbidity and mortality with reduced surgical time and length of ICU stay. Peripheral cannulation does not cause problem and cosmetic results were excellent.

